# Oxidative Stress and Skeletal Muscle Function

**DOI:** 10.3390/ijms241210227

**Published:** 2023-06-16

**Authors:** Guglielmo Duranti

**Affiliations:** Laboratory of Biochemistry and Molecular Biology, Department of Movement, Human and Health Sciences, Università degli Studi di Roma “Foro Italico”, Piazza Lauro de Bosis, 6, 00135 Rome, Italy; guglielmo.duranti@uniroma4.it; Tel.: +39-0636733479

Skeletal muscle is continuously exposed during its activities to mechanical/oxidative damage. Normal activity induces physiological adaptations that allow the muscle to counteract oxidative stress, but intense activity can have negative repercussions at a cellular and functional level. Excessive stretching and intensive exercise can cause a rupture of myofibril filaments, leading to the loss of skeletal muscle function through the failure of the excitation–contraction coupling system. These events generate an inflammatory response and a higher reactive oxygen species (ROS) production. ROS are continuously generated in the body and are usually promptly inactivated by the cellular antioxidant defenses. Low ROS concentrations in skeletal muscle modulate cell signaling processes and are required for normal force production. In contrast, higher ROS concentrations can lead to DNA, lipid, protein, and carbohydrate modifications, which can cause cellular function impairment and reduced force production, thereby contributing to muscle fatigue. For these reasons, the assessment of the impact of exercise at both the molecular and the biochemical levels, as well as its effect on cellular signaling pathways, constitute crucial points of interest for developing training protocols that are compatible with the health of different individuals.

In recent years, the number of scientific papers focusing on the study of muscle and its physiopathology has grown significantly [1]. This is not surprising, given that this tissue is fundamental to the well-being of the individual in many ways, such as for posture, movement, and metabolism. If we consider only the last twenty years, the increase in studies on this subject has been even more marked. Recently, particular attention has also been paid to the study of ROS production and its involvement in muscle pathophysiology (Figure 1) [2].

This Special Issue, “Oxidative Stress and Skeletal Muscle Function”, of the *International Journal of Molecular Sciences*, includes a total of seven contributions: six original articles and one review providing new information about the impact of oxidative stress on muscles under normal and pathological conditions.

There is a continuous and growing demand for strategies and/or supplements that can alleviate the negative effects of ROS produced by intense and vigorous physical activity at a systemic and general level (Figure 2) [3]. In particular, oxidative damage can compromise the functionality of the muscle tissue and, therefore, its sporting performance, especially in particular groups of subjects such as athletes. In this context, supplementation with antioxidant molecules is the most practiced activity (Figure 3) [4].

Generally speaking, these practices aim to protect the muscle cells from exercise-induced oxidative damage or improve recovery after their performance so as to allow the athlete to perform better. Antioxidant molecules of natural origin have proven effective in many contexts of exposure to oxidative stress [5,6,7].

Park et al. have demonstrated that the polyphenol Phloroglucinol showed strong antioxidant activity, protecting C2C12 muscle cells from H_2_O_2_-induced oxidative stress. Like other polyphenols, this molecule has been shown to enhance Nrf2/HO-1 signaling activity, thus activating the antioxidant enzymatic cellular system [8]. This cell defense mechanism is particularly important because it allows cells to adapt and better defend themselves against oxidative stress. 

Skeletal muscles have an efficient capacity for repair and regeneration in response to mechanical/oxidative injury. However, this process can be compromised in several pathological conditions such as myopathies, muscular dystrophy, and sarcopenia, conditions under which the capacity of the muscle regeneration process is impaired by oxidative stress. The activation of enzymatic antioxidant defense systems is, therefore, essential in these conditions. In particular, superoxide dismutase (SOD) activity protects against oxidative stress in the body. In mammals, there are three SOD isoforms: cytoplasmic SOD1, mitochondrial manganese SOD (SOD2), and extracellular copper/zinc-SOD (SOD3). SOD1 is considered a major isoform, since it is expressed as a predominant isoform in all cells [9].

In this context, the study of Takahashi et al. provides the first evidence that SOD1 reduction and the following superoxide overproduction delay skeletal muscle regeneration. This negative phenomenon is due to the induction of overt inflammation and fibrosis, especially in pathological conditions such as advanced progressive diabetic nephropathy (DN) [10]. Satellite cells are particularly sensitive to oxidative stress [11]. Quiescent satellite cells are activated to repair skeletal muscle damage undergoing active proliferation, and then differentiate into myoblasts and regenerate myofibers. The proper functioning of the enzymatic antioxidant system of these cells is, therefore, crucial in normal conditions and even more in pathological conditions. In fact, satellite cells are negatively regulated in conditions such as progressive DN [10].

Physical exercise induces a modulation of the cytokine/inflammatory profile. The muscle damage induced by intense activity causes a series of adaptations, including the cytokine/myokine release that stimulates the regeneration process. At the same time, the action of the immune system induces the production of local and systemic inflammatory events, which can compromise muscle activity and recovery after exercise [12]. As such, implementing strategies that allow the muscle to recover after the activity, which also decreases the inflammatory response, is equally important.

Jureka et al. demonstrated how a simple and minimally invasive technique, such as whole-body cryostimulation (WBC), is effective in this context. Using well-trained athletes, Jureka et al. proved that a single WBC session effectively modulated the cytokine profile after intensive exercise. This may suggest that even a single exposure to very low temperatures may normalize the body’s inflammatory response to exercise [13]. This effect is statistically supported by the correlations between the level of cytokines and oxidative stress markers in the blood of these subjects, both after leaving the cryochamber and after the exercise preceded by WBC. This could corroborate the relationship between ROS production and the modulation of the inflammatory response that normally occurs after vigorous exercise. This response, which can sometimes be negative on recovery from muscle damage, could be mitigated using this type of strategic approach [13].

Among reactive oxygen species, hydrogen peroxide (H_2_O_2_) exerts a critical regulatory role in skeletal muscle functions. In fact, low to moderate ROS levels are critical for cell signaling and the regulation of gene expression, inducing cell adaptations that are necessary for muscle growth [14]. On the other hand, high levels of ROS may lead to the formation of oxidized macromolecules that may contribute to the loss of myoblast function, increase myoblast cell death, and worsen muscle repair. These phenomena are especially evident in aging and/or pathological conditions that are associated with metabolic diseases such as diabetes or muscle mass loss, such as atrophy and sarcopenia [14].

It is now well known, with studies at the molecular level, that skeletal muscle adapts to the oxidizing environment generated by physical exercise by modulating enzymatic activities and producing useful metabolites at a local and systemic level.

For example, Antinozzi et al. demonstrated that moderate levels of H_2_O_2_ were able to induce modulation of steroidogenesis in muscle cells, as indicated by the increased expression of 3-HSD, 17-HSD 5-R2, and aromatase, as well as by the release of dihydrotestosterone (DHT) (but not testosterone (T)) [15]. DHT is a metabolite of testosterone that is produced in many tissues following the rapid and irreversible reduction in testosterone by 5-reductase. The effect of H_2_O_2_ on muscle cells found by Antinozzi et al. supports the hypothesis that DHT, but not T, shows a protective effect against oxidative stress. This has an evident repercussion on the physiology of the muscle cells by, for instance, the modulation of force production and an increase in muscle metabolic capacity. Antinozzi et al. hypothesize that DHT release can contribute to the adaptation process to an oxidizing environment, such as that caused by strenuous exercise, and that it probably ameliorates cell survival, considering the data from the literature [16].

Another interesting aspect of the adaptation of muscle cells to the action of H_2_O_2_ is that highlighted by Ceci et al. [17].

As previously mentioned, a central feature of the skeletal muscle is its ability to regenerate through the activation of satellite cells by environmental signals. 

Therefore, the negative action of ROS on these cells is crucial for the muscle regeneration process. Polyamines are among the various molecules that show the potential to support cellular replicative activity. Several studies have demonstrated the fundamental role of polyamines in maintaining muscle homeostasis and its physiological well-being [18]. Polyamines seem to play a role in protecting cells from ROS in different cell systems, and it has also been shown that the depletion of endogenous cellular polyamines results in increased sensitivity to ROS produced by radiation exposure [19].

Ceci et al. demonstrated that spermidine (Spd) treatment has a protective role in skeletal muscle cells by restoring redox balance (reducing the oxidized glutathione (GSH/GSSG) ratio) and increasing cell viability through a reduction in cell death. Moreover, Spd administration promotes recovery from culture-damaging conditions, such as a wound scratch assay. Taken together, all these features demonstrated that polyamines, and spermidine in particular, are able to make myoblasts more able to cope with an oxidative insult [17].

The important role of myoblasts and factors involved in muscle regeneration is also the subject of the interesting work of Miquel Perelló-Amorós et al. [20]. The process of myogenesis involves mesenchymal stem cells that are committed to the muscle lineage as myoblasts, undergoing a process that involves proliferation and cell fusion events. This complex cellular process is finely regulated by a series of myogenic regulatory factors (MRFs), which coordinate the expression of the required molecular machinery and structural components of the muscle. 

This complex myogenic program always occurs during the life of the individual from the embryonic stage to adulthood, and can be modulated in response to challenging conditions or tissue damage. Miquel Perelló-Amorós et al. demonstrated that important factors such as Myomaker and Myomixer play a crucial role not only during developmental myogenesis, but also when the myocyte differentiation takes place (particularly evident during late myogenesis), especially during regenerative myogenesis. Interestingly, both Myomaker and Myomixer genes in humans have a broad expression through a large list of cell types and tissues, and in particular, Myomaker is critical in coordination with other factors, such as MyoD and Myogenin, in regulating the transcription of Myomixer by binding to the specific three E-boxes of its promoter during myogenesis, activating the process.

Finally, Wei and Cui proposed a fascinating review highlighting the progress of knowledge of pyroptosis in muscle tissue [21].

Pyroptosis represents a newfound special cell death form directly related to programmed inflammation that exerts a protective role in the innate immune response by removing intracellular pathogens through the inflammatory reaction. This causes, as a consequence, the inhibition of intracellular pathogen replication and the activation of immune cells, thus promoting the trapping and elimination of pathogens.

Interestingly, pyroptosis may be involved in the inflammatory response and muscle injury induced by strenuous exercise and/or atrophic mechanisms [22]. On the other hand, aerobic exercise and resistance training may modulate pyroptosis in skeletal muscle cells, and this could represent an interesting new role of exercise in counteracting pathological conditions such as insulin resistance (IR). In fact, in IR, the impairment of the insulin signaling pathway by the inhibition of insulin or insulin-like growth factor 1 signaling, the consequent downregulation of PI3K/Akt expression, and the decrease in protein synthesis and FoxO1 (a transcription factor that regulates glucose metabolism, fat generation, and bone mass) phosphorylation can stimulate protein degradation through the activation of the ubiquitin–proteasome system, thus causing muscle injury and loss in diabetic patients, which also leads to muscle atrophy. Hence, the activation of the inflammasome and the inflammatory reaction have been confirmed to be involved in the pathological mechanism of skeletal muscle IR. In this context, inhibiting the inflammasome activation or blocking pyroptosis pathways can reduce the inflammatory responses and cell damage, relieving skeletal muscle IR.

Wei and Cui suggested that aerobic exercise and resistance training are able to improve pyroptosis in skeletal muscle IR, highlighting the anti-inflammatory function of exercise in skeletal muscle and consequently proposing exercise as a non-drug intervention in pathological conditions such as IR [21]. 

Altogether, the data presented in the papers published in the Special Issue “Oxidative Stress and Skeletal Muscle Function” of the *International Journal of Molecular Sciences* provide important new results regarding the complex process of skeletal muscle adaptation to oxidative stress and the complex mechanism of regeneration of skeletal muscle mediated by satellite cells. Detailed knowledge of these mechanisms is essential to better understand the physiopathology of muscle tissue, especially under disabling conditions that could lead to the degeneration of skeletal muscle and the consequent disability of the subject. In this context, it is essential to find new strategies to improve the well-being of patients and for the identification of new targeted therapeutic strategies.

## Figures and Tables

**Figure 1:**
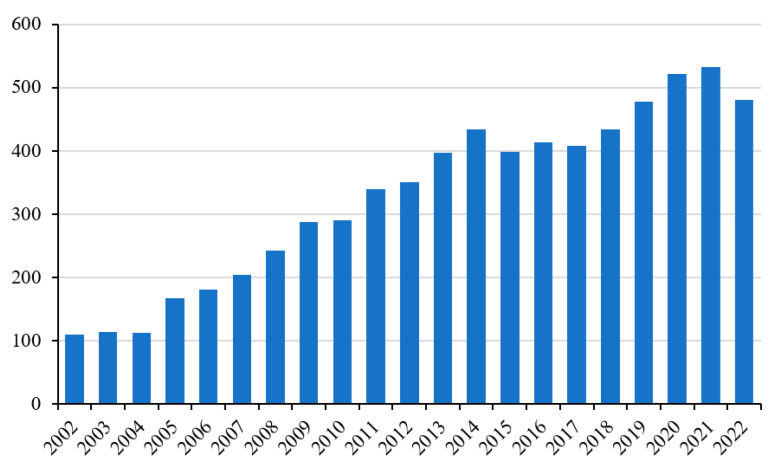
Number of publications indexed in PubMed from 2002 to 2022 applying keyword “skeletal muscle” in combination with “oxidative stress”.

**Figure 2:**
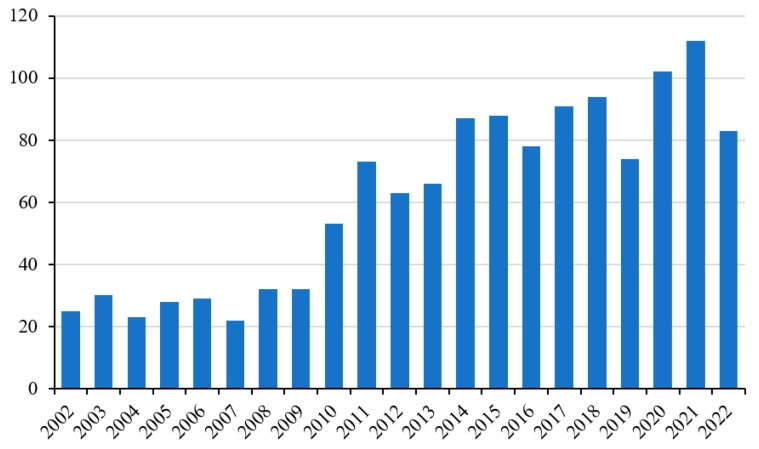
Number of publications indexed in PubMed from 2002 to 2022 applying keyword “skeletal muscle” in combination with “antioxidant supplementation”.

**Figure 3:**
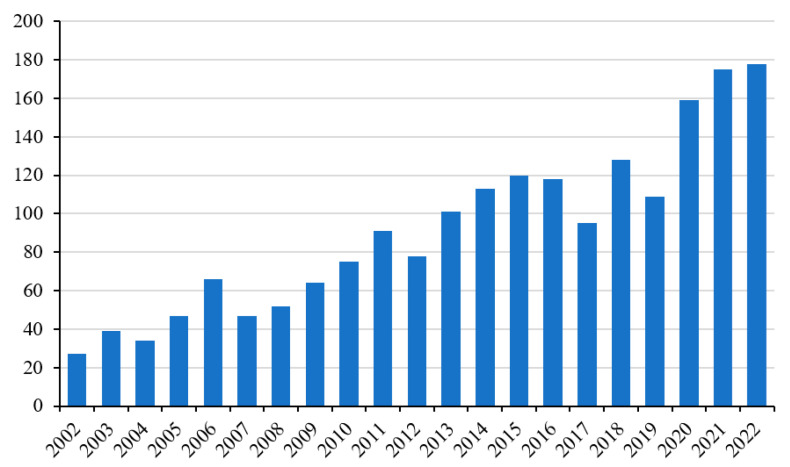
Number of publications indexed in PubMed from 2002 to 2022 applying keyword “exercise” in combination with “antioxidant supplementation”.

## References

[1] Duranti G. (2022). MUSCLES journal: The new home of muscle followers. Muscles.

[2] PubMed Website. https://pubmed.ncbi.nlm.nih.gov/?term=skeletal+muscle+oxidative+stress&filter=dates.2002%2F1%2F1-2022%2F12%2F31.

[3] PubMed Website. https://pubmed.ncbi.nlm.nih.gov/?term=skeletal+muscle+antioxidant+supplementation&filter=dates.2002%2F1%2F1-2022%2F12%2F31.

[4] PubMed Website. https://pubmed.ncbi.nlm.nih.gov/?term=exercise+antioxidant+supplementation&filter=dates.2002%2F1%2F1-2022%2F12%2F31.

[5] Ceci R., Maldini M., Olson M.E., Crognale D., Horner K., Dimauro I., Sabatini S., Duranti G. (2022). Moringa oleifera leaf extract protects C2C12 myotubes against H_2_O_2_-induced oxidative stress. Antioxidants.

[6] Tanabe Y., Fujii N., Suzuki K. (2021). Dietary Supplementation for Attenuating Exercise-Induced Muscle Damage and Delayed-Onset Muscle Soreness in Humans. Nutrients.

[7] Canals-Garzón C., Guisado-Barrilao R., Martínez-García D., Chirosa-Ríos I.J., Jerez-Mayorga D., Guisado-Requena I.M. (2022). Effect of Antioxidant Supplementation on Markers of Oxidative Stress and Muscle Damage after Strength Exercise: A Systematic Review. Int. J. Environ. Res. Public Health.

[8] Park C., Cha H.J., Hwangbo H., Ji S.Y., Kim D.H., Kim M.Y., Bang E., Hong S.H., Kim S.O., Jeong S.J. (2023). Phloroglucinol Inhibits Oxidative-Stress-Induced Cytotoxicity in C2C12 Murine Myoblasts through Nrf-2-Mediated Activation of HO-1. Int. J. Mol. Sci..

[9] Park J.H., Elpers C., Reunert J., McCormick M.L., Mohr J., Biskup S., Schwartz O., Rust S., Grüneberg M., Seelhöfer A. (2019). SOD1 deficiency: A novel syndrome distinct from amyotrophic lateral sclerosis. Brain.

[10] Takahashi Y., Shimizu T., Kato S., Nara M., Suganuma Y., Sato T., Morii T., Yamada Y., Fujita H. (2021). Reduction of Superoxide Dismutase 1 Delays Regeneration of Cardiotoxin-Injured Skeletal Muscle in KK/Ta-Ins2Akita Mice with Progressive Diabetic Nephropathy. Int. J. Mol. Sci..

[11] Bronisz-Budzyńska I., Kozakowska M., Pietraszek-Gremplewicz K., Madej M., Józkowicz A., Łoboda A., Dulak J. (2022). NRF2 Regulates Viability, Proliferation, Resistance to Oxidative Stress, and Differentiation of Murine Myoblasts and Muscle Satellite Cells. Cells.

[12] Markus I., Constantini K., Hoffman J.R., Bartolomei S., Gepner Y. (2021). Exercise-induced muscle damage: Mechanism, assessment and nutritional factors to accelerate recovery. Eur. J. Appl. Physiol..

[13] Jurecka A., Woźniak A., Mila-Kierzenkowska C., Augustyńska B., Oleksy Ł., Stolarczyk A., Gądek A. (2023). The Influence of Single Whole-Body Cryostimulation on Cytokine Status and Oxidative Stress Biomarkers during Exhaustive Physical Effort: A Crossover Study. Int. J. Mol. Sci..

[14] Sies H. (2017). Hydrogen peroxide as a central redox signaling molecule in physiological oxidative stress: Oxidative eustress. Redox Biol..

[15] Antinozzi C., Duranti G., Ceci R., Lista M., Sabatini S., Caporossi D., Di Luigi L., Sgrò P., Dimauro I. (2022). Hydrogen Peroxide Stimulates Dihydrotestosterone Release in C2C12 Myotubes: A New Perspective for Exercise-Related Muscle Steroidogenesis?. Int. J. Mol. Sci..

[16] Kajihara T., Tochigi H., Prechapanich J., Uchino S., Itakura A., Brosens J., Ishihara O. (2012). Androgen signaling in decidualizing human endometrial stromal cells enhances resistance to oxidative stress. Fertil. Steril..

[17] Ceci R., Duranti G., Giuliani S., Rossi M.N., Dimauro I., Sabatini S., Mariottini P., Cervelli M. (2022). The Impact of Spermidine on C2C12 Myoblasts Proliferation, Redox Status and Polyamines Metabolism under H_2_O_2_ Exposure. Int. J. Mol. Sci..

[18] Ha H.C., Sirisoma N.S., Kuppusamy P., Zweier J.L., Woster P.M., Casero R.A. (1998). The natural polyamine spermine functions directly as a free radical scavenger. Pro. Natl. Acad. Sci. USA.

[19] Amendola R., Cervelli M., Tempera G., Fratini E., Varesio L., Mariottini P., Agostinelli E. (2014). Spermine metabolism and radiationderived reactive oxygen species for future therapeutic implications in cancer: An additive or adaptive response. Amino Acids.

[20] Perelló-Amorós M., Otero-Tarrazón A., Jorge-Pedraza V., García-Pérez I., Sánchez-Moya A., Gabillard J.C., Moshayedi F., Navarro I., Capilla E., Fernández-Borràs J. (2022). Myomaker and Myomixer Characterization in Gilthead Sea Bream under Different Myogenesis Conditions. Int. J. Mol. Sci..

[21] Wei H., Cui D. (2022). Pyroptosis and Insulin Resistance in Metabolic Organs. Int. J. Mol. Sci..

[22] Hu S., Wan X., Li X., Wang X. (2022). Aerobic exercise alleviates pyroptosis-related diseases by regulating NLRP3 inflammasome. Front. Physiol..

